# PROTOCOL: Language of instruction in schools in low‐ and middle‐income countries: A systematic review

**DOI:** 10.1002/cl2.1319

**Published:** 2023-04-03

**Authors:** Pooja Nakamura, Zelealem Leyew, Adria Molotsky, Varsha Ranjit, Kevin Kamto

**Affiliations:** ^1^ International Development Division American Institutes for Research Washington District of Columbia USA; ^2^ Department of Linguistics Addis Ababa University Addis Ababa Ethiopia

## Abstract

To address the evidence gap in making effective language of instruction (LOI) decisions, we propose a systematic review of the role of LOI choices in education programs and policies on literacy outcomes in multilingual educational contexts in low‐ and middle‐income countries (LMICs). Grounded in a multidisciplinary theory of change (ToC) describing what factors link LOI choices and literacy outcomes, we will gather, organize, and synthesize the evidence on the specific role of the three LOI choices described in the ToC (teaching in mother tongue [MT] with later transition, teaching in a non‐MT language, or teaching in two or more languages at one time) and its impact on literacy and biliteracy outcomes. We will focus our systematic review and meta‐analysis only on quantitative and qualitative intervention studies from LMICs as these have the highest relevance for decision making in multilingual LMIC contexts. We will also only include languages that are relevant and commonly spoken in LMICs. For example, we will likely include studies that examine Arabic to English transfer, but not Arabic to Swedish transfer.

## 1 BACKGROUND

### 1.1 The problem

Even though more children are in school than ever before, more than 250 million of them are not learning basic literacy and numeracy skills (World Bank, 2018, 2019). Several factors contribute to this state of learning poverty, ranging from the macro (national education and teacher education systems) to the micro (cognitive learning mechanisms). However, one factor central to learning, but often under researched, misunderstood, or overlooked, is the role of language in education. The language the child is taught in is closely linked to successfully acquiring academic and socioemotional skills. Yet, language of instruction (LOI) programmatic and policy choices are made arbitrarily in many countries and are also constantly shifting based on community demand, political realities, and donor policies.

Children across low‐ and middle‐income countries (LMICs) are learning in multilingual contexts. This has wide‐ranging social, economic, political, and educational consequences for learning. Reading research makes it clear that children will learn to read only language(s) they understand (Hoover & Tunmer, 2020; NICHD Early Child Care Research Network, 2005; Ouellette, 2006). Even if children learn to decode (sound out) words—with some degree of fluency—reading comprehension will remain unattainable without sufficient oral language comprehension.

A few recent systematic reviews highlight the importance of instruction in the mother tongue (MT) (or a language the child speaks and understands well) for quality learning outcomes in LMIC's (Evans & Acosta, 2020; Nag et al., 2019). The benefits to MT‐based multilingual education programs are multifaceted, including higher likelihood of girls and marginalized communities staying in school (Benson & Hakuta, 2005), increasing educational equity and maintenance of cultural and linguistic diversity (Ball, 2010), allowing parents and communities to participate in the learning process (Nag et al., 2019) as well as long‐term cost benefits (Heugh, 2004, 2012). There are also clear cognitive benefits to learning to read in a known or familiar language, as the skills from the first language transfer and facilitate learning to read in a new language (Chung et al., 2019; Koda, 2008). Furthermore, strong bilingual education models have significant positive effects on non‐linguistic functions (Bialystok, 2005) and executive function skills (Bialystok, 2018) that lay a strong foundation for later socioemotional skills, as well as on academic achievement (Collier & Thomas, 2017).

At the same time, there is an ever‐increasing demand from communities for education in the national or international postcolonial language (Coleman, 2011). The primary reason for this demand is the link between the postcolonial language and socioeconomic mobility (Azam et al., 2010). Other factors that complicate LOI choices include linguistically heterogenous classrooms, in which there are multiple MTs in one school or area (Nakamura et al., 2017; Reddy, 2011); as well as the fact that some MT languages have no scripts, lack teaching and learning materials, have limited trained teachers, or lack political or community will to be implemented as languages in education (Piper et al., 2016; Trudell & Piper, 2013).

This leads to a situation in which decision‐makers must reconcile *both* the well‐documented benefits of MT instruction along with the quest for socioeconomic mobility through a postcolonial or international (later acquired) language at earlier grades. Therefore, this systematic review will focus on LOI choices in education programs and policies on student literacy outcomes in multilingual contexts in LMICs. In particular, we ask the question of whether or not MT (or familiar language) instruction impacts reading outcomes, as well as aim to investigate the unanswered question of when to introduce or transition to additional languages of instruction to foster quality bilingual or multilingual reading outcomes.

### 1.2 Theory of change for LOI policies and programs on literacy outcomes

#### 1.2.1 Theory underlying bilingual and multilingual literacy acquisition

To base our theory of change in theory (Brown, 2020), we developed a learning science^1^ framework of the *cognitive mechanisms* that underpin literacy learning in bilingual and multilingual learning contexts. This provides a theoretical basis for how we expect LOI policy and program interventions to impact literacy outcomes in LMIC's.

The Cognitive Foundations of Reading and its Acquisition (CFRA) is a model that lays out the cognitive components required for successful reading in *monolingual learners*—and links those components to curriculum effectiveness and reading teacher knowledge and teaching effectiveness (Hoover & Tunmer, 2020). The Peter Effect in teaching reading is rooted in the principle that it is not possible to give what one doesn't have (Applegate & Applegate, 2004; Binks‐Cantrell et al., 2012). Studies show that reading teacher effectiveness is significantly related to their own reading enthusiasm (Applegate & Applegate, 2004) as well as their own knowledge of the cognitive foundations of reading (Binks‐Cantrell et al., 2012).

Learning science theories from various disciplines such as psychology and linguistics reveal that the underlying mechanisms of acquisition of reading skills in *bilingual and multilingual learners* is different from learning to read in monolingual learners in significant and predictable ways. First, learning to read in a second or later acquired language (referred to henceforth as L2/x) is significantly impacted by transfer of reading skills from a first language (L1)^2^. Second, L2/x learning is also significantly impacted by L2/x oral language skills, which are highly variable in L2/x learners compared to monolingual learners. This notion that L2/x reading skills are reliant on a combination of L1 reading skills and L2/x oral language skills is encapsulated in the linguistic interdependence hypothesis, the underlying proficiency hypothesis (Cummins, 1979, 1981) and the transfer facilitation model (TFM) of second language reading (Koda, 2005, 2007, 2008).

Chung et al. (2019) provide an updated interactive framework for crosslinguistic transfer in L2/x reading, in which they posit that the relationship between L1 and L2/x reading skills is influenced by cognitive, linguistic, and metalinguistic factors such as language specific versus language neutral constructs, L1–L2/x distance, L1–L2/x proficiency and complexity. They extend the model to postulate that transfer is also impacted by socio‐cultural factors such as age of beginning acquisition of the L2/x, immigration experience, educational settings, and extent of exposure to the L1 and L2/x.

Indeed, empirical evidence is accumulating for each of these factors. In a meta‐analysis of the cognitive and linguistic sub‐skills in transfer, Melby‐Lervåg and Lervåg (2011) find that phonological awareness and decoding skills present significant correlations across L1–L2/x; but that these relationships are less (or not) present in oral language comprehension and reading comprehension subskills. Reflecting this need to start with a foundation in the L1 for successful outcomes in the L2/x, Collier and Thomas (2017) present the results from a 32‐year longitudinal research study on bilingual education in the United States. The results reveal that it takes an average of six years of high‐quality instruction in both the MT/L1 as well as English/L2/x, with at least 50% of the instruction being conducted in MT/L1 for English learners to perform comparably with their monolingual peers on academic outcomes.

There are increasingly diverse research methods being employed to answer a variety of question related to biliteracy development and LOI transitioning policy and practice questions. For example, studies have recently begun using threshold methodologies to examine ‘how much’ of a particular skill or knowledge is needed to benefit from transfer in biliteracy or bilingualism. In Northern England, De Cat et al. (2018) utilize Cox proportional hazard regression models to identify a threshold of ‘bilingual experience’ for early executive functioning skill benefits. In North America in a two‐way Spanish–English bilingual immersion program, Feinauer et al. (2017) employ discontinuous change‐point regression models to show that the relationship between L2 oral language skills and L2 reading development is not linear. Finally, in two LMIC contexts—India and Ethiopia—Nakamura and colleagues used structural break regression analyses to test whether there is an empirically determinable point of ‘sufficiency’ in the L1 literacy skills to transfer and foster success in literacy skills in the L2/x (Nakamura et al., 2018; Nakamura et al., 2019). The results showed that there was a nonlinearity in the relationship between first and second language reading scores in six languages pairs across these two countries, implying that there may be a point at which children are cognitively and linguistically ready for literacy instruction in L2/x as L1 reading skills reach a point of sufficient maturity for transfer to take place.

Beyond the cognitive underpinnings of biliteracy acquisition, certain aspects of the home environment are also identified as significant predictors of literacy and biliteracy acquisition. According to the Home Literacy Environment (HLE) model, informal language and literacy practices at home, that is, those not directly related to engaging with print at home are predictive of concept of print and emergent literacy skills; whereas, formal language and literacy practices at home, that is, those that are explicitly meant to teach children language and literacy skills are predictive of early decoding development (Senechal & Le Fevre, 2002, 2014). Cross‐country reviews also highlight that parental attitudes towards reading, number of books at home (indirect HLE factors) and literacy‐linked activities at home (direct HLE factors) have a significant impact on reading outcomes (Park, 2008). Given the vast mismatches between home and school language in LMIC's (Nag et al., 2019), as well as the generally lower rates of adult literacy (and thus parental literacy) in LMICs (Abadzi, 2003), the evidence underscores the additional risk of home literacy and language environments that do not have the resources necessary to support reading development in the language of the school—or any language (Nag et al., 2019). However, studies also suggest that there is context‐specificity in the relative importance of various dimensions of the home language and literacy environment on specific reading component outcomes (Friedlander, 2020; Nag et al., 2019; Park, 2008)

Classroom and teacher factors such as attendance (of both teachers and students), the lack of a safe learning space (Spier et al., 2019), nutritional inputs (Plaut et al., 2017), and availability of print (through digital media or not) are necessary factors for learning—however, they are not sufficient (Snilstveit et al., 2016). Pedagogical inputs (such as structured learning progressions, skill‐based learning, teaching and learning at the ‘right’ level) and teacher professional development are emerging as the most effective ingredients for translating access and safe learning spaces into quality learning outcomes (Evans & Acosta, 2020; Evans & Popova, 2015). In fact, Evans and Acosta (2020) underscore MT instruction programs as one of the most effective elements of pedagogical interventions.

Individual differences, such as age and socioeconomic status also are known to moderate the relationship between teaching inputs and language and literacy outcomes. Although it is clear that language learning abilities decline as individuals get older (Flege et al., 1999), there does not seem to be any conclusive evidence that there is a biologically based point at which a child's (or individual's) ability to learn a new language diminishes at a significantly higher rate than before such a point (Bialystok & Miller, 1999; Bialystok, 1991; Birdsong & Molis, 2001; Hakuta et al., 2003). Neurobiological studies reveal that age of acquisition does not alter the underlying brain structure of bilinguals (Frenck‐Mestre, 2016; Friederici et al., 2002). However, more intuitively, differential aspects of language learning (such as phonological and grammatic processing) are more susceptible to age‐associated declines than others, such as semantic processing (Abutalebi et al., 2001; Hernandez et al., 2000; Weber‐Fox & Neville, 2001). De Cat et al.'s (2018) study showed that socio‐economic status (SES) was a significant correlate of bilingualism's positive impacts; but found that the threshold effect of bilingual proficiency held even after SES was controlled for. In the United States and Canada, several evaluations of bilingual education program impacts on learning outcomes shows that although SES is a significant correlate of educational attainments, when controlled, bilingual children in bilingual programs outperform bilingual children in monolingual programs (Bialystok, 2018). Researchers in the West stress the critical difference between ‘bilingual education’ (positive, additive connotation) and ‘education of bilingual children’ (negative, subtractive connotation) (Bialystok, 2018). This distinction manifests itself in policies that either embrace bilingualism and multilingualism as a force for global integration versus those that use local language as steppingstones towards a different national or international language, which in turn contributes to motivation to learn and community and family involvement in the education system.

Taken together, these studies help us move towards the development of a middle range theory on multilingual education and biliteracy acquisition. However, it is still unclear how to construct an effective LOI policy, beyond noting that teaching a child in a language they are familiar with is critical for learning. There is little understanding of the mechanism of transfer of skills from one language to another, the ‘right’ timing or skill level at which a child is most likely to benefit from learning a new language, or how to foster quality bilingual/multilingual outcomes after the initial year(s) of MT instruction. As such, the policy question remains at what grade or point to transition students from one medium of instruction to another, and how to develop LOI decisions in LMIC's that are likely to be most impactful in improving literacy and biliteracy scores. It is also unknown whether such an LOI model is likely to be the same across different contexts. It is thus critical to consolidate evidence through a rigorous evidence synthesis.

#### 1.2.2 Intervention logic framework

Based on the problem statement above and the theoretical framework of bilingual/biliteracy learning, it is clear that L1 and L2 skills are significantly related in complex and constantly interacting ways that are important for the development of an intervention logic framework. As such, there are many ways that language transition interventions that aim to improve literacy skills may be effective.

**Figure 1:**
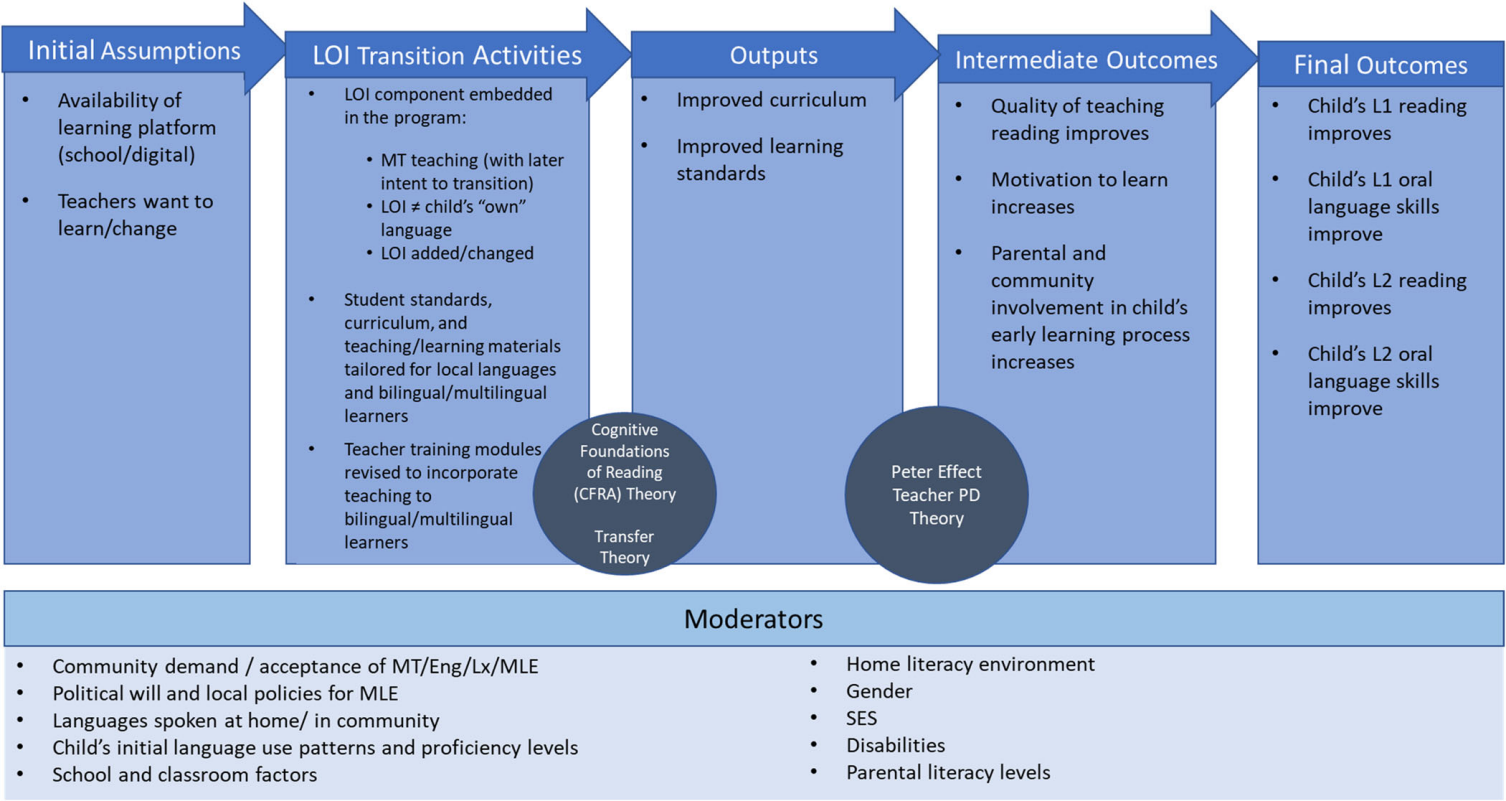
Logic framework for language of instruction (LOI) policies and interventions on literacy outcomes.

In Figure 1, we present our logic framework. We begin with the key assumption that the child has access to a learning program. Access can occur in the form of school infrastructure with teachers who do not use any technology, or a blended learning environment within a school or community building where teachers or teaching assistants may use some technology to enhance learning (e.g., the eSchool 360 model implemented by the Impact Network in Zambia), or an online/digital learning program that is—or could be—facilitated by a remote teacher or guide (e.g., Mindspark software), or an entire online/digital learning program that is self‐guided or guided by a virtual built‐in guide (e.g., the Google Bolo app). Another assumption underlying our logic framework is that teachers are willing and able to learn and change how they teach in line with new curricula, teaching materials, and pedagogies tailored to bilingual or multilingual students and varying language types. The introduction of revised LOI choices cannot be effective or adequately applied within the classroom without a revision in the teaching and learning materials to reflect this change. Further, since not all teachers may be fully fluent in the LOI choices or know how to teach those language(s), some may be required to obtain additional trainings to improve teaching knowledge or be re‐assigned to schools where they can teach language(s) they are fluent in and trained to teach reading in.

LOI transition intervention activities can be manifested in many ways. For the purposes of this research, we operationalize LOI transition programs and policies as those that have any one or any combination of the following components:

(1) An education program or policy that is implemented in a MT or local language, which will then lead to a transition to (complete change) or addition of (adding as a subject or dual language instruction) teaching of a new language, which may or may not occur during the course of the program. These are important to examine as the skills taught and learned during the course of the program will have significant implications for ‘readiness’ of transfer to the new later acquired language.
(2) An education program or policy that is implemented in a language that is not the child's ‘own’ language (i.e., a language the child has enough proficiency to learn in). These programs are important to investigate as they constitute a child ‘transitioning’ out of their own language into an education system in a new language right from the start of education.
(3) An education program or policy that is implemented in which students transition from one LOI to another or add a new language as part of the medium of instruction during the course of the program or policy.

In any of these scenarios students’ learning acquisition process can be impacted by the language used for teaching—and as such, can have significant implications for effectiveness of learning to read. This is regardless of whether the LOI transition is the key component of the educational program.^3^

Furthermore, the program or policy intervention is most likely to succeed in improving learning outcomes if it has standards, curriculum, and trained teachers (the latter for classroom‐based instruction, as opposed to technology‐based instruction) that focus on the cognitive foundation models (CFRA) (Hoover & Tunmer, 2020) and/or the interactive models of reading transfer (Chung et al., 2019). Although several programs may not explicitly have these theoretical frameworks named in their models, this is based on the theoretical premise that a curriculum or a teacher cannot give what they do not have (Applegate & Applegate, 2004; Binks‐Cantrell et al., 2012).

These programmatic components (or activities) may improve the quality of teacher (or technology) knowledge and practices, increase child's motivation to learn (as this will maintain teaching at the ‘right level’, Banerjee et al., 2016; Pritchett & Beatty, 2015), and also increase parental and community involvement in the child's education (Benson & Hakuta, 2005). Finally, all these will improve the effectiveness of the LOI decision, leading to impacts on the child's L1 literacy skills (reading or decoding based skills as well as oral language skills) and the child's L2/x literacy skills.

We also examine the role of several possible factors that are likely to moderate the likelihood that the intervention will improve literacy skills, including community demand for the L1 versus the L2/x, local and national policies supporting the implementation of the program or policy, socioeconomic status, parental literacy/schooling level, language(s) spoken at home, home literacy environment (exposure to print), child's initial language use and proficiency level in language(s) of the school, gender, and disability.

Given that LOI choices touch several aspects of the education system, we aim to synthesize the linkages between the inputs to trace how components of LOI programming may impact different sections of the system. For instance, inputs in curricular choices in terms of timing and sequencing of skills in each language would impact standards and curriculum development decisions; whereas teacher training inputs would impact professional development modules, assignment of teachers to schools based on languages they speak versus languages students speak, and urban‐rural teacher availability. All practice and policy recommendations will be interpreted within the theory of change, to further develop a middle‐range theory for LOI decision making in LMICs that is reflective of both the micro psycholinguistic and learning science ingredients in improving learning outcomes as well as the macro sociolinguistic, socioeconomic, and political environment within which LOI policy and practice decisions are being made.

## 2 WHY IT IS IMPORTANT TO DO THE REVIEW

This systematic review will aim to help decision makers—ministries of education, teacher training institutes, community leaders, interested donors, and implementing and research organizations—understand and effectively use existing evidence related to multilingual education. Most reading programs being funded by large international donors such as USAID and UNICEF include programming in the MT for at least the first few years of primary school (e.g., the USAID‐funded Creative‐implemented Vamos Ler! project in Mozambique or READ II program in Ethiopia, or the USAID‐funded RTI‐implemented PRIMR program in Kenya). Yet, countries continue to shift policies at the national level (e.g., Rwanda switched from a Kinyarwanda LOI policy to an English only policy in 2019 reversing a 2015 MT only policy reform, Edwards, 2019), and regional governments continue to change the years of transition of medium of instruction (e.g., India's regional push towards regional language education despite community demands for sooner English medium of education, Amaravati, 2019; Gejji, 2019). Indeed, recent international education reports continue to emphasize that ‘learning poverty’ cannot be solved without a better evidence base on how to tackle the role of LOI in multilingual LMIC educational settings (World Bank, 2018, 2019).

We will also examine evidence gaps that may hinder efforts to implement successful LOI policies in multilingual LMICs. We will focus particularly on the ministry of education in Ethiopia to guide the implementation of the country's new three language policy that is part of the education roadmap reform being developed for rollout in the near future. Our conversations with key stakeholders in the Ethiopian education system, including the Ministry of Education team that is tasked with developing the roadmap development suggest there is an urgent need to gather and understand the evidence on how to implement this multilingual LOI policy effectively.

The generalizability of the findings across language types is informed by the framework that all writing systems of the world are divided into four main types (Nag & Perfetti, 2014; Perfetti, 2003): alphabetic, syllabic, alphasyllabic, and morphosyllabic. Although we will examine various local contextual factors that may hinder or facilitate the impact of the program, including linguistic complexities on the dimensions of orthographic depth, orthographic breadth, graphic complexity in both/all languages, we will be able to consolidate the findings for a broader middle‐range theory for each of the four writing system types.

This study builds on recent systematic reviews that have shown that MT instruction is critical for learning quality (Evans & Acosta, 2020; Nag et al., 2019); but is unique in that it will be the first to systematically review the evidence on how and when to add—or transition from—one LOI to another. Furthermore, the study utilizes a combination of methods, including critical discourse analyses to map policy and practice documents to the evidence generated from the systematic review. Finally, by exploring various psycholinguistic underpinnings of reading and examining a variety of sociolinguistic contexts of learning and drawing from our multi‐disciplinary theoretical framework, it helps build middle‐range theory on the mechanisms that may explain why certain LOI policies are likely to be more effective than others.

## 3 OBJECTIVES

To address the evidence gap in making effective LOI decisions, we propose a systematic review^4^ of the role of LOI choices in education programs and policies on literacy outcomes in multilingual educational contexts in LMICs. Grounded in the multidisciplinary theory of change described above of what factors link LOI choices and literacy outcomes, we will gather, organize, and synthesize the evidence on the specific role of the three LOI choices described in the ToC (teaching in MT with later transition, teaching in a non‐MT language, or teaching in two or more languages at one time) and its impact on literacy and biliteracy outcomes. We will focus our systematic review and meta‐analysis only on quantitative and qualitative intervention studies from LMIC's as these have the highest relevance for decision making in multilingual LMIC contexts. We will also only include languages that are relevant and commonly spoken in LMIC's. For example, we will likely include studies that examine Arabic to English transfer, but not Arabic to Swedish transfer. Against this backdrop, we pose the following research questions:

***Primary Research Questions**:*

1. What are the short‐ and long‐term impacts of **initial LOI** choices on literacy and biliteracy outcomes, and how do they differ across various LMIC contexts?
2. What are the short‐ and long‐term impacts of **LOI transition** on literacy and biliteracy outcomes, and how do they differ across various LMIC contexts?
***Secondary Research Question**:*
3. What are the overall messages of key donor and stakeholder agencies around LOI choices and learning outcomes? How do those messages link to the existing evidence?
***Additional Mapping Questions**:*
4. What is the quality of the available evidence on the role of LOI choices on literacy outcomes?
5. What are the evidence gaps about the role of LOI choices in bilingual and multilingual educational contexts in LMICs?

## 4 METHODOLOGY

In this section we provide detail on the methods that will be employed to answer **our primary research question**.

### 4.1 Criteria for including and excluding studies

#### 4.1.1 Types of study designs

The primary research question on the effectiveness of interventions will be addressed using quantitative experimental or quasi‐experimental as well as qualitative studies that include a programmatic or policy intervention.

Specifically, we will include the following study designs for **quantitative studies**: (1) experimental designs using random assignment to the intervention and (2) quasi‐experimental designs with nonrandom assignment (such as regression discontinuity designs, ‘natural experiments’, and studies in which participants self‐selected into the program). Quasi‐experimental studies must (1) collect longitudinal data (baseline and end line) or cross‐sectional data (end line) from treatment and comparison groups and (2) use propensity score or another type of matching, difference‐in‐differences estimation, instrumental variables regression, multivariate cross‐sectional regression analysis, or other forms of multivariate analysis (such as the Heckman selection model or multivariate ordinary least squares regression analysis). We will include studies with data collected at the individual level to ensure that the study focuses on child‐level learning outcomes.

We will include each of the multivariate quasi‐experimental methods to maximize the external validity of the systematic review. However, several of the quasi‐experimental studies we propose to include may include only OLS regression analysis and, therefore, may not be able to provide unbiased impact estimates. In such cases, we exclude these studies from our meta‐analysis. To mitigate concerns about internal validity of some of the included studies, we will conduct a risk of bias assessment and stratify our meta‐analysis by identification strategy, where feasible, as in Brody et al. (2015). This stratified meta‐analysis will enable us to assess the internal validity of the included studies with a high risk of bias by comparing the impact estimates in those studies with the impact estimates in studies with a low risk of bias (Chinen et al., 2017).

For **qualitative studies**, we will include any intervention studies that utilize the following illustrative methods: (1) case studies, (2) focus group discussions; (3) key informant interviews; and (4) observations of classrooms or community language use. At the abstract screening stage, we intend to include all qualitative studies that examine an intervention, regardless of methodology. Depending on the number of studies that are returned from the abstract screening stage, we will either select only those qualitative studies that are linked to a quantitively examined intervention, to explain why or how that particular intervention may or may not be effective; or select all qualitative intervention studies for full‐text review, which will provide a fuller picture of how and why multilingual or MT instruction programs may or may not be effective in LMIC's in general.

#### 4.1.2 Types of participants and settings for both quantitative and qualitative studies

We will include studies that focus on interventions that include primary and secondary school aged children in LMICs, as defined by the World Bank.^5^ We will include studies about the effects of LOI choices regardless of the educational status or skill level of children at the time of the intervention. Only studies conducted between the years of 1995 and 2020 and published in English or Amharic will be considered.

In the case of qualitative studies, we will include intervention studies that have a focus on school‐aged children in LMICs, as defined by the World Bank.

#### 4.1.3 Types of interventions

The interventions included in this review will be LOI choices made by educational policies and programs that *directly* aim to increase children's literacy in bilingual or multilingual LMIC education contexts. These interventions include programs with one or more of the following components:

- Full early learning programs for MT education or bilingual and multilingual children
- Official LOI policy (laws or de facto policy) changes
- Teacher training for MT programs or bilingual or multilingual education programs^6^
- Standards development for MT or bilingual or multilingual education programs
- Technology‐based interventions for MT or bilingual or multilingual education programs
- Mother tongue or bilingual teaching and learning materials
- Mother tongue or bilingual books
- Mother tongue or bilingual book clubs, libraries, community reading spaces, mobile book vans, and so forth.
- Assessments used as part of MT of bilingual programming
- Mother tongue or bilingual or multilingual radio or media programming

#### 4.1.4 Types of comparison conditions

Eligible comparison conditions will include no intervention, pipeline, or ‘business as usual’. Where the typical comparison condition of ‘no intervention’ or ‘business as usual’ is not selected, eligible comparison conditions will include students before a LOI policy change within a region, students within regions with different or no LOI transition policies within a country, and students in schools who are not in a MT or regional language program. In the case of qualitative studies, a comparison will not be necessary.

#### 4.1.5 Types of outputs and outcome measures

We will include studies that focus on intermediate and/or final outcomes. Below we present definitions for each of the output and outcome variables for the primary research question:

***Outputs***:

1. **Curriculum and Standards**: We define the curriculum as a document or framework that spells out a wide range of content, objectives, methodologies, resources, assessments, and organizational information regarding what a child is expected to learn. A curriculum should be closely aligned with content standards (what students should know and be able to do at a particular grade) and performance standards (a scale to indicate how, and what percentage of students are performing on the content standards). These standards in turn are of high quality when they are linked to the cognitive foundations of reading acquisition (Hoover & Tunmer, 2020).

***Intermediate Outcomes***:

1. **Reading teacher quality**: Based on the Peter Effect studies that a reading teacher cannot pass on what they themselves do not have, we define two main dimensions in reading teacher quality: (a) the enthusiasm to teach reading as indicated by the habits and practices of the teacher (Applegate & Applegate, 2004) as well as (b) the knowledge of the sub‐constructs required for learning to reading (Binks‐Cantrell et al., 2012).
2. **Student motivation**: Defined as a child's motivation to attend school as well as to want to learn to read or engage with language, print, or stories. This will be measured in student's reading behaviours at home or in school and in the community, and attitudes towards reading.
3. **Parental and community involvement**: Studies have found that there is a significant association between student's being taught in home and community languages and the parental involvement in the education system (Benson, 2007). We define this outcome as the frequency and quality of interactions between the parents and/or community members and teachers, as well as the amount of time spent by teachers involved with student's learning at home (helping with homework, learning *from* the student, supporting the student with their learning, asking questions about school, etc.)

***Final Outcomes***:

We provide operational definitions for our final outcomes from a range of research on reading development that looks at reading across language and orthographic types as well as across L1/L2 learning status (Koda & Zehler, 2008; USAID et al., 2019; Verhoeven & Perfetti, 2017). Each of these skills will be considered for both L1 and L2/x:

1. **Emergent literacy sub‐skills**: Emergent literacy skills or ‘concept of print (or print awareness)’, refer to the ability to understand the conventions and functions of print in one's own language(s), including the ability to distinguish pictures from letters or words, identify the beginning and ending of stories, and so forth.
2. **Oral language skills**: These skills include a range of receptive and expressive oral language abilities, such as the ability to follow simple spoken instructions, identify the meaning of spoken word, explain the meaning of a word in their own words, retell a short story, understand explicit and implicit comprehension questions from a short listening passage, and so forth.
3. **Metalinguistic awareness**: Metalinguistic awareness is the awareness of the functionally useful units of speech and print and the relationship between the two. There are several sub‐components of metalinguistic awareness, but we will focus on **phonological awareness**, or the awareness of speech units in any language and **morphological awareness**, or the awareness of morphological units in any language.
4. **Sound‐symbol correspondences**: Oftentimes called letter naming or letter knowledge, sound‐symbol correspondence skills refer to the ability to see a single printed letter, akshara, or character and be able to sound out the symbol.
5. **Decoding**: The ability to see a printed word or cluster of symbols and sound out the word or cluster of symbols. There are several paths that learners take to acquire this skill, but our primary concern will be on whether or not students are able to reach the entire phonological representation of the printed word, regardless of which path they take to achieve the skill.
6. **Oral reading fluency**: The ability to sound out a short passage or story with accuracy, speed, and prosody.
7. **Reading comprehension**: The ability to comprehend both explicit and implicit information presented in single or multiple phrases or sentences of text.

If data are not available for each of these subskills separately, based on the CFRA (Hoover & Tunmer, 2020), we will create composite scores for the emergent literacy and oral language measures (#1–3 above), and for the decoding scores (#4–6 above), and for the reading comprehension scores (#7). Understanding that there are usually only four questions on many EGRA scores for reading comprehension, we will consider either removing these items or merging them with the decoding scores for reliability, if necessary.

We will include only literacy outcomes even if the study looks at scores on other subjects.

Outcome measures will not be considered to filter qualitative studies, which will serve to address the secondary research question.

### 4.2 Search strategy

We developed a search strategy in consultation with an information specialist. Our search strategy will enable us to identify relevant published and unpublished literature by focusing on relevant academic and institutional databases, citation tracking, and snowballing of references. We identified the following literature searches.

### 4.3 Electronic sources

Comprehensive database searches will include the following paid‐access and free‐access electronic databases:

#### 4.3.1 Paid‐access databases

1. ERIC (Education Resources Information Center)
2. Education Source
3. EdWorkingPapers
4. EdArXiv
5. Registry of Efficacy and Effectiveness Studies (REES)
6. PsycINFO
7. JSTOR Arts & Sciences I‐X Collections
8. JSTOR Business III Collections
9. SAGE Publications
10. ScienceDirect
11. Springer Science+Business Media
12. Taylor & Francis
13. Wiley
14. WorldCat

#### 4.3.2 Open‐access databases

15. Campbell Collaboration
16. Cochrane Library
17. 3ie Impact Evaluation Repository
18. Directory of Open Access Journals (DOAJ)
19. Directory of Open Access Books (DOAB)
20. Development Experience Clearinghouse (DEC)
21. Institute of Development Studies (eldis)
22. Education International

#### 4.3.3 Grey literature

Grey literature searches will include a review of institutional websites as well as generic searches such as via Google and Google Scholar. In addition to the following institutional websites, key papers will be examined in search of other relevant institutional sources.

#### 4.3.4 Institutional websites or research funders

1. The UK Department for International Development (DFID) (https://www.gov.uk/government/organisations/department-for-international-development)
2. The US Agency for International Development (USAID) (https://www.usaid.gov/)
3. The Joint Libraries of the World Bank and International Monetary Fund (JOLIS) (https://library.worldbankimflib.org/)
4. J‐PAL (http://www.povertyactionlab.org)
5. UNESCO (https://en.unesco.org/)
6. UNICEF (https://www.unicef.org/)
7. UNICEF Office of Research (https://www.unicef-irc.org/)
8. UNHCR (https://www.unhcr.org/)
9. Population Council (https://www.popcouncil.org/)
10. World Vision (https://www.worldvision.org/)
11. Save the Children (https://www.savethechildren.org)
12. Plan International (https://plan-international.org/)
13. Organization of American States (OAS) (http://www.oas.org/en/)
14. Global Partnership for Education (GPE) (https://www.globalpartnership.org/)

Forward and backward snowballing of the references of key papers will provide additional studies for review that may not be found in database searches. Citation searches will be conducted in Google Scholar, Scopus, and Web of Science. This set of key papers will include:

#### 4.3.5 Key papers

15. Anchor key papers identified by the authors. These papers are listed below.
16. Key papers identified by external funder (CEDIL).
17. Studies that pass the inclusion criteria.
18. Additional key papers identified from institutional websites.

Table 1 presents the list of key papers identified by authors and reviewers to be used in citation searches.

**Table 1:** Key papers.

Study
Piper, B., Zuilkowski, S., & Ong'ele, S. (2016). Implementing mother tongue instruction in the real world: Results from a medium‐scale randomized controlled trial in Kenya. *Comparative Education Review, 60*, 776–807.
Brunette, T., Piper, B., Jordan, R., King, S., & Nabacwa, R. (2019). The impact of mother tongue reading instruction in twelve Ugandan languages and the role of language complexity, socioeconomic factors, and program implementation. *Comparative Education Review, 63*, 591–612.
Banerji, R. Berry, J., & Shotland, M. (2017). The impact of maternal literacy and participation programs: Evidence from a randomized evaluation in India. *American Economic Journal: Applied Economic, 9(4)*, 303–337.
Chicoine (2019). Schooling with learning: The effect of free primary education and mother tongue instruction reforms in Ethiopia. *Economics of Education Review, 69*, 94–107.
Laitin, D. D., Ramachandran, R., & Walter, S. (2019). The legacy of colonial language policies and their impact on student learning: Evidence from an experimental program in Cameroon. *Economic Development and Cultural Change, 68 (1)*, 239–272.
Shin, J., Sailors, M., McClung, N., Hoffman, J. V., Pearson, D., Kaambankadzanja, D., & Mwale, L. (2019). Access to local books: The effects of Read Malawi from a children's rights perspective.

#### 4.3.6 Search strings

We developed search strings in collaboration with an information specialist. Each search string is adapted to fit the syntax of the database host to utilize Boolean operators (AND/OR), wildcards, truncation, and other database search features. The search strings are designed to return studies that include at least one keyword in the following four themes:

1. Participants: preschool, elementary school, preprimary, kindergarten, primary school, early childhood.
2. Literacy: reading, literacy, MT, LOI, medium of instruction, cross‐language transfer, language transition, reading transfer, multilingual education, bilingual education.
3. Setting: low‐income, middle‐income, third world, developing, underdeveloped, LMIC, global south, Africa, Asia, LAC, Southeast Asia.

To capture both quantitative and qualitative literature relevant to the primary research questions, we will not include search strings for study design, comparison condition, and outcome measures. Using these criteria in the search strategy would exclude relevant qualitative studies in addition to quantitative and mixed‐methods studies that omit this information from the title and abstract. We will assess the inclusion criteria for study design, comparison condition, and outcomes during the screening of the studies.

### 4.4 Screening

Screening will take place in two phases: first on the basis of titles and abstracts and then on the basis of full texts.

#### 4.4.1 Screening phase 1

After our initial search is completed, we will conduct a manual abstract review process. Each abstract will be reviewed independently by two trained reviewers. We will conduct the following steps in the abstract screening phase (based on Polanin et al., 2018).

1. Creation of the abstract screening tool. The tool has been created taking into consideration the following issues:
  a. Questions are objective and ‘single‐barrelled’
  b. Questions include yes/no/don't know structures, with dropdown options as needed
  c. Detailed key for dropdown options (such as what is meant by ‘literacy outcome’ or ‘school‐age’)
  d. Questions are ordered hierarchically to ensure that studies that are not meeting any one inclusion criteria are immediately removed from further screening
2. Review of the tool by the lead of our quantitative analysis
3. Train reviewers on the use of the tool
4. Conduct pilot tests of using the screener with the review team all screening the same 15–20 abstracts until consensus is reached on the tool.
5. Periodic check‐ins to enhance intellectual buy‐in as well as to ensure any disagreements are resolved

#### 4.4.2 Screening phase 2

In the second phase, we will review the full text of all studies that pass Phase 1 screening. Multiple reviewers will independently identify and confirm the following information for each study:

#### 4.4.3 Quantitative studies

- Target population
- Intervention type
- Comparison group
- Quantitative methodology
- Data source
- Outcome measures

#### 4.4.4 Qualitative studies

- Target population
- Intervention type
- Qualitative methodology
- Gender norms
- Barriers to intervention effectiveness
- Facilitators of intervention effectiveness

Studies pass Phase 2 screening if the information pulled from each study passes the inclusion criteria listed in the previous section.

## 5 METHODOLOGY TO SYNTHESIZE THE LITERATURE

### 5.1 Quantitative studies

#### 5.1.1 Data extraction

Two team members with expertise in quantitative research will work independently to extract information from each quantitative study included in the review. Both team members will use a data extraction form and fill the data from the extraction form in a table. We will resolve disagreements through discussion.

#### 5.1.2 Risk of bias assessment

We will determine the rigour of the quantitative studies using an adaptation of a set of criteria, to assess risk of bias in experimental and quasi‐experimental studies (Hombrados & Waddington, 2012). While the risk of bias assessment is very labour intensive, the number of quantitative studies we expect to require this assessment is low. We will assess the risk of the following biases:

1. Selection bias and confounding, based on quality of identification strategy to determine causal effects and assessment of equivalence across the beneficiaries and nonbeneficiaries.
2. Performance bias, based on the extent of spillovers to students in comparison groups and contamination of the control or comparison group.
3. Outcome and analysis reporting biases, including:
  a. The use of potentially endogenous control variables
  b. Failure to report nonsignificant results
  c. Other unusual methods of analysis
4. Other biases, including:
  a. Motivation and courtesy biases (Hawthorn effect and John Henry effect)
  b. Coherence of results
  c. Retrospective baseline data collection
  d. Differential attrition bias
  e. Other biases, such as strong researcher involvement in the implementation of the intervention.

#### 5.1.3 Measures of treatment effects

In accordance with, Chinen et al. (2017); we will extract information from each quantitative study to estimate the standardized effect sizes (for continuous variables) or odds ratios (ORs) (for binary variables) across studies. We will also calculate standard errors and confidence intervals where feasible.

We will report effect sizes as Hedge's *g* and will adjust effect sizes reported as Cohen's *d* to Hedge's *g*. We will use Hedges’ *g* effect sizes (sample‐size‐corrected standardized mean differences [SMDs]) for continuous outcome variables, which measure the effect size in units of standard deviation of the outcome variable, and for binary outcomes, we will calculate ORs.

The SMD using Cohen's *d* is calculated by dividing the mean difference with the pooled standard deviation by applying the formula in the following equation:

(1) $$\mathrm{SMD}=\frac{Y_{t}-Y_{c}}{S_{p}},$$

where *Y*_*t*_ refers to the outcome for the treatment group, *Y*_*c*_ refers to the outcome for the comparison group, and *S*_*p*_ refers to the pooled standard deviation.

The pooled standard deviation *S*_*p*_ will be calculated by applying either of the following equations:

(2) $$S_{p}=\frac{\sqrt{\left(\left({{\mathrm{SD}}_{y}}^{2}\right)\times \left(n_{t}+n_{c}-2\right)\right)-\left(\frac{\beta^{2}\times (n_{t}\times n_{c})}{n_{t}+n_{c}}\right)}}{n_{t}+n_{c}},$$

(3) $$S_{p}=\frac{\sqrt{\left(n_{t}-1\right)\times s{{}_{t}}^{\begin{array}{c} 2\\ \; \end{array}}+\left(n_{c}-1\right)\times {s_{c}}^{2}}}{n_{t}+n_{c}-2},$$

where *SD*_*y*_ refers to the standard deviation for the point estimate from the regression, *n*_*t*_ refers to the sample size for the treatment group, *n*_*c*_ refers to the sample size for the comparison group, and *β* refers to the point estimate. We will use Equation (2) for regression studies with a continuous dependent variable and Equation (3) when the study provides information about the standard deviation for the treatment group and the comparison group.

To transform Cohen's *d* into Hedge's *g*, we will use the small sample correction for the SMD by applying on the following formula:

(4) $${\text{SMD}}_{\text{corrected}}={\text{SMD}}_{\text{uncorrected}}\times \left(1–\frac{3}{4\times \left(n_{t}+n_{c}-2\right)-1}\right).$$

Lastly, Equation (5) will estimate the standard error of the SMD:

(5) $$SE=\sqrt{\frac{n_{t}+n_{c}}{n_{c}\times n_{t}}+\frac{{\mathrm{SMD}}^{2}}{2\times (n_{c}+n_{t})}}.$$

For studies using linear probability models, we will assume linearity in the estimation of standardized effects as in Brody et al. (2015). For example, if we observe a mean baseline value for the comparison group of 0.097 and an effect size of 5.1 percentage points, then we will assume that the follow‐up value for the treatment group would be 0.097 + 0.051 = 0.148, and we will assume that the follow‐up value for the comparison group will be 0.097. To correct the standard errors for studies where the outcome variable is clustered at a level above the individual or household, we will use adjusted standard errors by applying corrections to the standard errors and confidence intervals using the variance inflation factor (Higgins & Green, 2011):

(6) $${\mathrm{SE}}_{\mathrm{corrected}\;}={\mathrm{SE}}_{\mathrm{uncorrected}}\times \surd (1+(m-1)\times \mathrm{ICC}),$$

where *m* is the number of observations per cluster, and ICC is the intracluster correlation coefficient. We will estimate the ICC for each of the relevant outcome measures for our sample of included quantitative studies and for which we are able to access the data on the outcome measures.

When we are unable to retrieve the missing data, we will impute effect sizes and associated standard errors based on the *t* or *F* statistic or *p* values. We will use David Wilson's practical meta‐analysis effect‐size calculator to conduct such imputations. Where sample sizes for the treatment and the comparison group are not reported, we will assume equal sample sizes across the groups.

#### 5.1.4 Methods for handling dependent effect sizes

Where studies report more than one effect size on the basis of different statistical methods, we will follow the procedure as laid out in Chinen et al. (2017) and will select the effect size with the lowest risk of bias. Where studies report more than one effect size based on the same individuals, we will employ the robust variance estimation techniques to adjust for effect size dependency (Hedges et al., 2010). When studies present multiple impact estimates for different variables measuring the same construct, we will use a sample‐size weighted average to measure a ‘synthetic effect size’. In cases where more than one study uses the same data set (e.g., national level EGRA data) to measure a literacy outcome, we will use the effect size from the study with the lowest risk of bias. If the risk of bias is the same, we will estimate an average effect size through inverse‐weighted random effect meta‐analysis. In cases where one study measures the same outcome at different points in time, we will extract the effect size by relying on the outcome measure that was measured closest to the time period of the measurement in other studies included in the same meta‐analysis. In cases where studies include more than one treatment arm, we will include the effect size from the treatment arm with the lowest risk of bias. If the risk of bias is the same, we will use the effect size from the treatment arm that is most similar to the other programs included in the meta‐analysis.

### 5.2 Meta‐analysis

We will pool the results of the quantitative studies that focus on the same outcome variables and same intervention types using meta‐analysis. In other words, we will conduct multiple different meta‐analyses based on intervention type and outcome variable (described above which reading outcomes will be pooled if necessary). We will examine the heterogeneity of the effect sizes for each outcome across studies and use meta‐regression to model the variation in effect size and will use forest plot visualization (Borenstein et al., 2009). We will use Stata to conduct the meta‐analysis.

For the meta‐analysis, we will include only studies with an emphasis on LOI choice that use one of the following designs: (1) experimental designs using random assignment to the intervention and (2) quasi‐experimental designs with nonrandom assignment (such as regression discontinuity designs, ‘natural experiments’, and studies in which participants self‐select into the program).

Where possible, we will perform sensitivity analysis for potential moderators:

- Risk of bias status for each risk of bias category;
- Study design (randomized controlled trials vs. quasi‐experimental studies);
- Gender
- SES
- Parental literacy levels
- Alignment of language spoken at home with LOI
- Geography

We will use random‐effects meta‐analysis because the average effect of LOI choice is likely to differ across contexts due to differences in program design and contextual characteristics. We will supplement our random‐effects meta‐analysis with network meta‐analysis to enable indirect comparisons of two treatments that have a common comparator.

We will also use stratified meta‐analysis according to contextual and methodological moderator variables to investigate factors explaining heterogeneity. We will use two contextual moderating variables: (1) type of orthography and (2) grade.

#### 5.2.1 Missing data

In cases where it is not feasible to estimate the effect size because of missing data, we will contact the researchers to request the missing information to calculate the effect sizes. If authors do not respond or do not provide sufficient information to calculate the effect size, we will not include the study in the meta‐analysis. Even so, the study and its findings will still be discussed within our narrative write‐up.

### 5.3 Treatment of qualitative research

Every study that is selected for full‐text review, will undergo a full quality appraisal.

#### 5.3.1 Quality appraisal

We will assess the quality of the qualitative studies using the nine‐item Critical Appraisal Skills Programme Qualitative Research Checklist (Critical Appraisal Skills Programme, 2013), judging the adequacy of stated aims, the data collection methods, the analysis, the ethical considerations, and the conclusions drawn. For each item, one trained researcher on our team will independently fill out the appraisal to determine whether the study had adequately met the item and gave ‘yes’, ‘no’, or ‘can't tell’ response. Afterwards, they will then come together to discuss their responses to each item until they reach consensus. We will rate studies that score 0–2 ‘no’ or ‘can't tell’ responses as low risk of bias, studies that score 3–5 ‘no’ or ‘can't tell’ responses as medium risk of bias, and studies that score 6–9 ‘no’ or ‘can't tell’ responses as high risk of bias (Table 2).

**Table 2:** Quality appraisal criteria

Criteria	Coding
*Screening Question*: Is there a clear statement of study aims of the research?	*Yes/Can't tell/No*
*Screening Question*: Is a qualitative methodology appropriate?	*Yes/Can't tell/No*
Is it worth continuing?	*Yes/Can't tell/No*
Was the research design appropriate to address the aims of the research?	*Yes/Can't tell/No*
Was the recruitment strategy appropriate to address the aims of the research?	*Yes/Can't tell/No*
Were the data collected in a way that addressed the research question?	*Yes/Can't tell/No*
Has the relationship between researcher and participants been adequately considered?	*Yes/Can't tell/No*
Have ethical issues been taken into consideration?	*Yes/Can't tell/No*
Was the data analysis sufficiently rigorous?	*Yes/Can't tell/No*
Is there a clear statement of findings?	*Yes/Can't tell/No*
Is the research valuable?	*Yes/Can't tell/No*

After full‐text review, we will conduct a thematic synthesis of the qualitative study findings. Each study's main findings will be coded to encapsulate the content of each findings (e.g., ‘the teacher joins Portuguese and the local language to help the student understand’, ‘we use [the local language] only to pull the student from where he is and understand the subject’). These statements are then categorized into higher order themes (such as ‘use of local language in post‐colonial language classes’). We will then extract implications for better understanding why or how multilingual education choices work in various contexts.

#### 5.3.2 Critical discourse analysis

To answer our secondary research question focused on the overall policy messages being conveyed by the key donor and stakeholder agencies around LOI choices, we will conduct a qualitative critical discourse analysis (CDA), through Systemic Functional Linguistics (SFL) analysis (including linguistic and textual analysis) and ideological analysis (Martin & Rose, 2007; Van Djik, 2006) on 2‐3 key donor and MOE documents on LOI policies or strategies.

By using CDA, we will analyse the discourse in these LOI documents, and the LOI‐related discourse that has been included and excluded by these donor institutions and understand the dynamics between development assistance network, donor institutions and, if feasible, nation‐states. In addition, we will conduct CDA based on discursive psychology to understand the positions and power relations between these donor institutions and nation‐states. The discursive nature helps explore the identity of stakeholders, their positions and their narration within a social context (Hajer, 1995; Hewitt, 2009). Using the discursive tradition will help reveal how donors justify certain models of MT based education and how donors persuade nation‐states on LOI policies. Together, the SFL and Ideological analyses approaches will help us identify how stakeholders present and/or consume a ‘shared set of ideas’ about language and education; for example, how is evidence discussed and applied, how are groups conducting MT education versus post‐colonial medium of instruction discussed and described, and so forth.

SFL provides a framework through which we will analyse linguistic features used: how often do they use particular words? What affect do those words carry? Do the linguistic features carry explicit or implicit power structures in terms of LOI choices and consequences? What type of evidence is used to justify certain models of LOI? How is the concept of ‘mother tongue’ described by the donor institution versus the MoE and on what narrative ideology is this concept based on? Given that labelling is one of the very first steps in realizing how language is treated in any language policy or planning decisions (Kaplan & Baldauf, 1997), and terminological variance abounds in LOI policies worldwide, this will also shed considerable light on the links between how donors, other decision makers, and the layman consume and use information and evidence on language decisions in education.

These approaches will allow us to select, analyse, and interpret the essential messages embedded in donor policies and discourse around language issues in education policies. The primary theoretical premise of CDA is that language choices are shaped by, and shape, society and that language policy is influenced largely by historical, social, political and ideological environments in nation‐states (Fairclough, 2009). As such, this approach will help various stakeholders—MOEs, teacher training institutes, implementing organizations—understand and to objectively evaluate donors’ monetary prioritizations; as well as for donors to review, and if necessary, revise or adapt their existing and future education policies to incorporate a more evidence‐based view of language issues.

Once the CDA has been conducted, we will disseminate the findings through webinars, blog posts, social media, and individual communications to key stakeholders, including MOE personnel and key members from donor organizations and implementing partner organizations. The results will be shared in such a way that they are tailored to each stakeholder through policy briefs, brochures, and easy to access materials. The dissemination will be closely followed by co‐interpretation and planning meetings or workshops. In these meetings, the stakeholders will be encouraged to closely analyse existing documents and discourse around the role of language in education—including assessing whether the discourse itself is absent—and then work together to determining changes that could likely steer the course of LOI policy in a direction more in line with the evidence, as well as highlight gaps that are still hindering decision makers’ abilities to move forward with more effective LOI policies and practices.

## 6 QUALITY ASSURANCE (QA)

AIR has a rigorous system for QA, which ensures that we deliver high‐quality products. Dr Thomas de Hoop serves as our QA reviewer on this project; he has over 13 years of experience designing and managing large‐scale systematic reviews in education in LMICs. He will be responsible for providing support to the team and for ensuring the quality of the research materials produced. Specifically, Dr. de Hoop will support the project team by providing inputs on the design of evidence synthesis’ protocol, refining work and analysis plans, reviewing analysis and results, making suggestions about interpretation of further analysis, and reviewing final research products including blogs, policy briefs, and the final synthesis report. Dr. de Hoop will sign off on all drafts and final drafts after QA of each deliverable.

### 6.1 COVID‐19 risk mitigation

AIR has extensive experience facing and overcoming challenges associated with managing and conducting research. While the desk‐based nature of the evidence synthesis greatly reduces the risks to the project, especially considering the current COVID‐19 pandemic, we are aware that a few potential risks remain. For instance, there is the possibility that one or more of our project team members may be directly or indirectly affected by COVID‐19, subsequently, reducing their ability to work on the project. However, we developed the project team such that every position has backup support from another staff member, so the project is unlikely to be delayed due to COVID‐19 affecting any one team member. If we experience the unfortunate circumstance of multiple team members being affected by COVID‐19 at the same time, this will require us to pull from our internal staffing networks to employ additional staff to support the project team with the research activities. Again, we do not foresee this resulting in any delays to the progress of the project.

## ROLES AND RESPONSIBILITIES

• Content: Pooja Nakamura, Zelealem Leyew
• Systematic review methods: Pooja Nakamura, Adria Molotsky, Varsha Ranjit, Kevin Kamto
• Statistical analysis: Adria Molotsky, Kevin Kamto
• Information retrieval: Pooja Nakamura, Zelealem Leyew, Adria Molotsky, Varsha Ranjit, Kevin Kamto
• Quality review: Thomas de Hoop

## SOURCES OF SUPPORT

This review is supported UK Aid through the Centre of Excellence for Development Impact and Learning (CEDIL).

## DECLARATIONS OF INTEREST

None of the proposed authors have developed impact studies that focus on the LOI transition policies.

## PRELIMINARY TIMEFRAME

**Table:** 

**Deliverable**	**Date to Submit**
Title Registration	June 2020
Protocol	July 2020
Draft review	May 2021
Final review	August 2021

## PLANS FOR UPDATING THE REVIEW

The authors will assess updating the review every three years if funding becomes available.

## Supporting information

Supporting information.

## Footnotes

1 Learning science is the field of study that aims to ‘better understand the cognitive and social processes that result in the most effective learning, and to use this knowledge to redesign classrooms and other learning environments so that people can learn more deeply and more effectively’ (Sawyer, 2006, p. xi).

2 We refer to L1 as the first language(s) of the child or any language(s) the child uses and understands with high levels of familiarity and proficiency, oftentimes called ‘mother tongue’ (MT). We refer to L2/x as the second or later acquired language(s) of the child, that is, any language(s) they are learning and have emerging levels of familiarity and proficiency with.

3 Where possible, we will isolate the impact of LOI from impacts of other programming features such as learning materials, teacher professional development, and so forth. However, the likelihood of finding studies within our search for which we are able to do so is low as LOI policies are rarely, if ever, randomly assigned or assigned in isolation.

4 We follow the definition of systematic reviews and evidence syntheses from the Campbell Corporation: ‘a systematic review is an academic research paper… that uses a method called evidence synthesis to look for answers to a pre‐defined question’.

5 We are using the 2014 LMICs definition, which includes Argentina, Hungary, Seychelles, and Venezuela. These countries, however, were categorized as high‐income countries in the July 2015 update.

6 Bilingual or multilingual programs are defined as any that have more than one language of instruction, have additional language subject classes, or the students are bilingual or multilingual, or students are in mixed‐language/linguistically heterogenous classrooms
