# Supplementary material for: PROTOCOL: Language of instruction in schools in low‐ and middle‐income countries: A systematic review
Source: Campbell Syst Rev. 2023 Apr 3;19(2):e1319. doi: 10.1002/cl2.1319 (PMC10068939; doi:10.1002/cl2.1319)
Supplement: Supplementary file 1 — Supporting information. [file CL2-19-e1319-s001.docx]

## Appendix 1: Example Search Strategy

| **Query** |
| --- |
| “Read*” or “Literac*” OR “Mother Tongue*” OR “First Language*” OR “Second Language*” OR “third language” OR “language of instruction” OR “medium of instruction*” OR “cross-language transfer” OR “cross-linguistic transfer” OR “language transition*” OR “L1-L2 transfer” OR “reading transfer” OR “cross-linguistic” OR “English language learners” OR “ELL*” OR “MTB-MLE” OR “multilingual education” OR “dual language*” OR “ESL” OR “English as a second language” OR “English as an additional language” OR “EAL” OR “Language Policy” OR “Bilingual Education” OR “postcolonial language” OR “colonial language” OR “English medium” OR “minority language*” OR “majority language*” OR “home language*” OR “language of the playground*” OR “community language*” OR “language of wider communication*” OR “language minority” OR “limited English proficiency” OR “Mother tongue based multilingual education” OR “dual language immersion” OR “translanguag*” OR “Language Transition” |
| “Preschool” OR “pre-school” OR “elementary school” OR “pre-primary” OR “kindergarten” OR “pre-k” OR “prek” OR “primary grade*” OR “primary school*” “early childhood” |
| “Low and middle income countries” OR “LMIC*” OR “develop* country” OR “global south” OR “Africa” OR “Asia” OR “LAC” OR “Southeast Asia” or “Latin America and the Caribbean” OR “Latin America |

## Appendix 2: Pilot Phase 1 Screening

If any of the preliminary exclusion criteria can be checked, the study is excluded and screening is stopped. For each of the criteria, do not exclude a study if there is not enough information in the title and abstract to exclude with certainty.

1. □ **Exclude** if date of publication is before 1995.
2. □ **Exclude** if language is not English or Amharic.
3. □ **Exclude** if setting is a high-income country.
4. □ **Exclude** if there is no reference to literacy, reading, or LOI.

Studies that remain are then assessed for their relevance to the primary or secondary research questions. Studies may be relevant to more than one of the research questions, for example, if they have a mixed-methods design or explore the secondary research question.

If a study is quantitative primary evidence, use the exclusion criteria that immediately follow. After the study is either included or excluded based on these criteria, proceed to screen the study on the basis of the second research question. If a study passes any of the three sets of inclusion criteria, it will be included in Phase 2 for full text screening.

1. □ Quantitative primary study (research question 1)

5a. □ **Exclude** if the study does not focus on evaluating an intervention.

5b. □ **Exclude** if the study does not use longitudinal or cross-sectional data collected at the individual level.

5c. □ **Exclude** if the study design is not experimental or if the study is quasi-experimental without propensity score or another type of matching, difference-in-difference estimation, instrumental variable regression, multivariate cross-sectional regression analysis, or other forms of multivariate analysis (such as the Heckman selection model or multivariate ordinary least squares regression analysis).

5d. □ **Exclude** if the study does not include a valid comparison condition, such as “no intervention,” “pipeline,” or “business as usual.”

5e. □ **Exclude** if the study does not include any of the following outcome measures.

5f. □ **Include** for research question 1 if the study passes all of the previous criteria.

1. □ Qualitative primary study (research question 2)

6a. □ **Exclude** if the study is not tied to specific LOI policy interventions.

6b. □ **Exclude** if the study does not report a qualitative methodology, such as interviews or focus groups.

6c. □ **Exclude** if the study does not include students.

6d. □ **Include** for research question 2 if the study passes all of the previous criteria.

1. □ **Include** for Phase 2 if any of 5f, 6d, or 7d are checked.
